# Contaminated Pond Water Favors Cholera Outbreak at Haibatpur Village, Purba Medinipur District, West Bengal, India

**DOI:** 10.1155/2014/764530

**Published:** 2014-05-12

**Authors:** Dilip Kumar Biswas, Rama Bhunia, Dipankar Maji, Palash Das

**Affiliations:** ^1^Health-II, District: Purba Medinipur, West Bengal, India; ^2^Lady Dafferin Victoria Hospital, Kolkata, West Bengal, India; ^3^Government of West Bengal, Swasthya Bhawan, Salt lake, Kolkata, West Bengal 700091, India; ^4^Department of Community Medicine, Midnapore Medical College, Paschim Medinipur, West Bengal, India

## Abstract

Health workers reported an increased number of diarrhea cases at Haibatpur village on June 17, 2012. This outbreak was investigated with the following *objectives*: to confirm the existence of diarrhea outbreak, to find out the risk factors, and propose control measures. Cases were listed; spot map and epidemic curve were drawn. Attack rate was calculated by age and sex and risk factors were found out by calculating odds ratio (OR) with 95% confidence interval (CI). Rectal swabs were taken and water specimens were collected for laboratory test. Forty-one cases of patients were identified with overall attack rate (AR) was 5% (41/780). AR among men was higher 6% (25/404) than women. There was no death. *V. cholerae* 01 Eltor Ogawa was isolated from one (1/4) stool specimen. Spot map showed cases clustered around two ponds which were contaminated with coliform organisms. The underground water was a bit saline in nature. Using pond water for preparation of fermented rice (Panta Bhat) (OR 4.73, 95% CI 1.69–13.51), washing utensil in pond water (OR 7.31, 95% CI 1.77–42.29) were associated with cholera outbreak. Health education was done to villagers. Disinfection of two ponds with bleaching powder was done. We proposed supplying of safe drinking water and repairing defective deep tube well to village.

## 1. Introduction

*Vibrio cholerae* are commonly found in seas, estuaries, brackish water, rivers, and pond water of coastal areas [1]. It is transmitted by contaminated water and food. Cholera is characterized by profuse watery diarrhea, vomiting, abdominal cramps, circulatory collapse, and shock [2]. Morbidity of cholera provides acute suffering, monitory loss, and probability of impending epidemic. As a consequence mortality due to cholera is observed. Surface water is frequently contaminated with* V. cholerae *and rural people often remain in close contact with surface water [3]. Surface water includes pond water, canal water, and river water. Rural people usually used these water for washing utensil and garments, bathing, and preparation of foods. These practices augment the transmission of diarrheal diseases. It was estimated in 2010 that 3–5 million people suffered from cholera and more than 100,000 people died due to cholera worldwide [2]. A total of 13,819 cholera cases were reported from 14 countries of Asia in 2010 with a 50% increase in the number of cases than 2009. In 2010, India accounted for 5,155 cholera cases [4]. Sixty-eight outbreaks of cholera which were reported in India during 1997 to 2006 affected more than 200,000 cases and 823 deaths [5]. In 2010, 192 cholera cases were reported in West Bengal [6]. Cholera is still a public health problem in West Bengal, India. Eleven diarrhea outbreaks were reported in the district of Purba Medinipur in 2011, of which none were confirmed using laboratory facilities. Health workers informed the Block Medical Officer of Health (BMOH) of Kharipukuria Block that there were increased numbers of diarrhea cases in Haibatpur village. The outbreak was investigated with the following* objectives*: to confirm the existence of the diarrhea outbreak, find out the risk factors, and propose control measures.

## 2. Materials and Methods

### 2.1. Descriptive Epidemiology

The subcenter and the diarrhea affected village were visited and reviewed for diarrhea case records of last three years. We looked for any population influx in the village and also checked for any change of case definition and surveillance system. A diarrhea case was defined as passing of three or more loose stools in a day among any resident of Haibatpur village between June 12, 2012, and June 30, 2012 [7]. A confirmed cholera case was defined as isolation of* V. cholerae* 01 or 0139 among diarrhea cases of any resident of Haibatpur village between June 12, 2012, and June 30, 2012 [7]. During the investigation, three types of dehydration were observed among the cases such as (i) no dehydration which includes not enough signs to classify some dehydration and severe dehydration and only ≥ three loose stools within the 24-hour period, (ii) some dehydration (any two of the followings) such as restlessness and irritability, sunken eyes, drinking eagerly and thirsty, and skin pinch which goes back slowly by less than two seconds, and (iii) severe dehydration (any two of the followings) such as lethargy and unconscious, sunken eyes, inability to drink, and skin pinch which goes back very slowly (taking more than 2 seconds) [8]. Cases were searched for by house-to-house survey of the affected village. Line-listing of cases was done noting name, age, sex, date of onset, signs and symptoms of diarrhea, and outcome of disease. The spot map was drawn to show the distribution of cases of patients. The attack rate was calculated by age and sex using the population denominator of the village. The epidemic curve was drawn to show the magnitude of outbreak. To generate hypothesis trawling questionnaires were administered among five diarrhea cases of patients. Epi-info and Excel software were used for analysis.

### 2.2. Laboratory Investigation

Four rectal swabs were collected from the cases that did not take any antibiotics and sent to the laboratory of School of Tropical Medicine, Kolkata, West Bengal, for physical, chemical, and cultural sensitivity test. Five water samples were also collected from different drinking water sources and ponds of the village and sent for examination to the Public Health Laboratory, Purba Medinipur, for physical, chemical, and bacteriological examination.

### 2.3. Environmental Study

The affected areas were inspected and observed for water supply system, drainage system, water storage conditions, kitchen articles, food preparation practices, and sanitary condition of the village cultural beliefs. Cases of patients and villagers were interviewed about source of drinking water, sanitation, and personal hygienic practices.

### 2.4. Analytical Study

Closed-ended questionnaires were used for data collection. Four trained health workers collected data. A case-control study was conducted to test the hypothesis that pond water was associated with the cholera outbreak. All the diarrhea cases were included as cases during the study period. We selected controls from next-door neighborhood. A control was defined as any resident of Haibatpur village who did not have ≥ three loose stools a day between June, 12, 2012, and June 30, 2012, above the age of five years. One case was compared with one next-door neighbor control. Panta Bhat is a lightly fermented rice-based dish consumed in Bangladesh and the Eastern Indian States of West Bengal and Assam. Panta means “soaked in water” and Bhat means “boiled rice.” This dish of leftover rice, soaked in water to prevent spoiling, is generally served with salt, onion, and chili. It is especially popular in rural areas served as a breakfast. Odds ratios (OR) were calculated with 95% confidence interval (CI).

## 3. Result and Discussion

### 3.1. Descriptive Epidemiology

**Figure 1:**
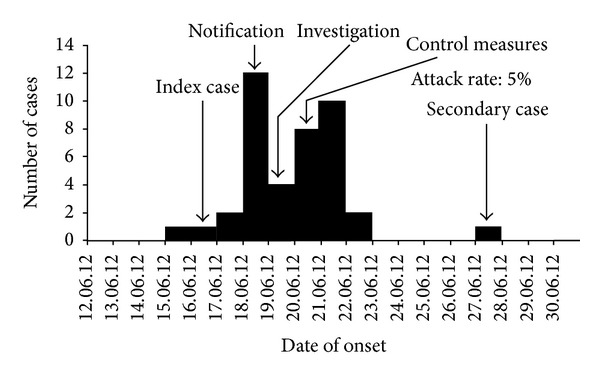
Epidemic curve of diarrhea outbreak at Haibatpur village, Purba Medinipur district, West Bengal, India, 2012.

**Figure 2:**
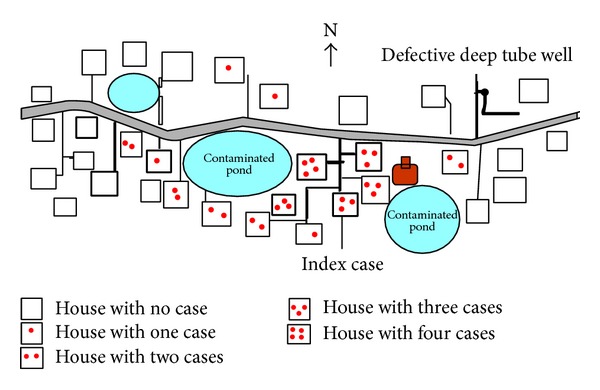
Spot map of diarrhea cases at Haibatpur village, Purba Medinipur district, West Bengal, India, 2012.

The population of the village was 780 [9], of which 703 were above the age of five years. The total diarrhea cases of same month of last three years were assessed through record review and a total 11 (3 + 4 + 4) cases were recorded. This year a total 41 cases were identified at the same month and this was clearly in excess of the expected number of diarrhea cases. The cases were reported between June 15, 2012, and June 28, 2012, a total of 14-day period with a peak on June 18 (Figure 1). Total forty-one cases were identified with no death (case fatality ratio = 0). Overall attack rate (AR) was 5% (41/780). Men, 6% (25/400), were affected more than women. AR was highest among the age group of >60 years, 9% (5/54), followed by 6% (26/264) among the age group of 15–59 years. No case was detected below the age of 5 years (Table 1). More than 80% (33/41) of cases developed no dehydration to some dehydration but 20% (8/41) of cases required intravenous infusion due to severe dehydration. Cases were clustered around two ponds (Figure 2).

### 3.2. Laboratory Result

*V*.* cholerae* 01 Eltor Ogawa was isolated from one stool specimen (out of four). Five water samples were tested (two pond water samples of the affected village and three tube well water samples from the adjacent villages) from where the local people collected water for drinking, of which two pond water samples were found contaminated with coliform organism above the maximum permissible number (MPN), indicating fecal contamination. All the three tube well water samples had no growth and physical quality was good.

### 3.3. Case-Control Study

All forty-one cases and same number of controls were recruited for the case-control study. Median age for case was 33 (range 5–80) years compared with the median age of controls which was 34 (range 6–75) years. Females were 31% and 53% among the cases and controls, respectively. Compared to control, cases more likely had used pond water samples for preparation of fermented rice (OR 4.73, 95% CI: 1.69–13.51), had used pond water for washing utensils (OR 7.31, 95% CI: 1.77–42.29), or had history of diarrhea case contact (OR 3.41, 95% CI: 1.25–9.47). Cases were less likely those who washed hands after defecation (OR 0.08, 95% CI: 0.02–0.31) (Table 2).

### 3.4. Environmental Study

There was scarcity of safe drinking water in this Gram Panchayat area due to defective deep tube well. The deep tube well was out of order for the last five months. Underground water was saline in nature. The villagers were compelled to consume pond water during preparing food, washing utensil, and other day-to-day activities. They collected drinking water from the nearby villages with the minimum distance of one to three kilometers. The cases were clustered around the pond and the hypothesis was generated that pond water was associated with the cholera outbreak. The local people prepared fermented rice (Panta Bhat) with pond water. The villagers said that the fermented rice would be tasty when it was prepared with pond water. Three days before the start of outbreak, the index case consumed fast food from the street vendor followed by diarrhea. Her soiled garments were washed in the nearby pond water. The villagers used this pond water for preparation of the fermented rice and washed their utensils and vegetables. The cases were distributed around two ponds of the village (Figure 2).

The diarrhea outbreak was probably due to cholera. Two pond water samples were found contaminated with fecal materials. There was scarcity of safe drinking water in the village. They used to collect safe drinking water from a distance of 1–3 kilometers. The villagers used contaminated pond water for preparation of fermented rice (Panta Bhat). The middle-aged and older men were affected mostly. The cases were clustered around two ponds.

The index case had the history of taking fast food from a street vendor. Study in Dhaka, Bangladesh, showed that cholera was transmitted through contaminated food served by street vendor and restaurant caterer [10]. Physical and chemical characteristics of food that supported the survival of* V*.* cholerae* O1 and O139, such as low temperature, high organic contents, increased moisture contents, neutral or alkaline pH Hydrogen ion concentration, pH 7 is neutral, <7 is acidic and >7 is alkaline. Fermented rice had the favorable conditions for survival and transmission of* V*.* cholera* [11].* V*.* cholerae* also can survive in cooked rice, potatoes, eggs, and pasta for more than five days and also in spices including pepper and cinnamon for several days [12, 13]. Several foods, fruits, vegetables, sea foods, diary product, poultry, and meat products act as a medium for transmission of* V*.* cholera* [14, 15].

The contaminated surface water was associated with cholera outbreak studied in Kenya [16]. The villagers usually used pond water for preparation of fermented rice that they consumed. The villagers also used this pond water for cooking and washing of the cooking utensils and vegetables. They used to take bath in these two ponds and had a habit of gurgling with this water during bath. The villagers close to these ponds were affected more than the distant area. A study in the state of Orissa revealed that well (shallow underground water source) became contaminated with* V*.* cholerae* and people close to the well were affected more than the distant area [17]. The outbreak of cholera usually occurs in the summer season. Warm weather is suitable for their spread and it is commonly transmitted through fecal-oral route [18]. In this summer season, there was shortage of water; a small amount of water was retained at the bottom of the ponds. Only four stool specimens were collected. We could not collect more than four stool specimens as cases had consumed antibiotics earlier.

## 4. Conclusion 

Contaminated pond water used for preparing fermented rice and other domestic uses facilitated cholera outbreak. The causative agent was* V. cholerae* 01 Eltor Ogawa. We recommended a few interventions. First, we educated the villagers not to use pond water till diarrheal outbreak was over. Second, immediate and periodic disinfection of pond water with bleaching powder was done. Third, we educated the villagers regarding the danger of use of pond water for preparing fermented rice, and benefit regarding washing hands before taking food and after defecation with soap with soap after defecation. Fourth, we recommended repairing the defective deep tube well and arrangement of piped water supply to the villagers. Fifth, we recommended oral cholera vaccine use in cholera endemic areas and research on cultural behaviors during cholera outbreak.

## Figures and Tables

**Table 1:** Age and sex specific attack rate of diarrhea cases at Haibatpur village, Purba Medinipur district, West Bengal, India, 2012.

Characteristics	Age groups	Cases of patients	Population	Attack rate (%)
Age in years	<5	0	77	0
	5–14	10	185	5
	15–59	26	464	6
	>60	5	54	9

Sex	Male	25	400	6
	Female	16	380	4

	Total	41	780	5

**Table 2:** Selected risk factors of the cholera outbreak at Haibatpur village, Purba Medinipur district, West Bengal, India, 2012.

Risk factors	Population				Odds ratio	95% CI*
	Cases		Controls	
	Number	%	Number	%
Pond water used for fermented rice preparation	30	73	15	37	**4.73**	1.69–13.51
Pond water used for washing utensils	38	93	26	63	**7.31**	1.77–42.29
History of diarrhea contact	29	70	17	42	**3.41**	1.25–9.47
No hand wash before eating properly	39	95	25	61	**12.48**	2.52–117.69
Tube well water used for drinking purpose	34	83	35	85	**0.83**	0.21–3.24
Open defecation practice	10	24	5	12	**2.32**	0.63–9.54
Hand washing practice after defecation with soap	18	44	37	90	**0.08**	0.02–0.31

*CI: confidence interval.
